# Spiritual care challenges and needs of carers, health professionals and relatives of individuals living with neurodegenerative diseases in palliative and end-of-life care: a scoping review protocol

**DOI:** 10.1136/bmjopen-2025-114259

**Published:** 2026-04-02

**Authors:** Enrico De Luca, Andreina Saba, Laura Bertarini, Antonio Brusini, Giovanna Artioli, Federica Dellafiore

**Affiliations:** 1 University of Birmingham, Birmingham, UK; 2 Department of Primary Care, Azienda USL - IRCCS di Reggio Emilia, Reggio Emilia, Italy; 3 Department of General and Post-Acute Internal Medicine, University Hospital Modena, Modena, Italy; 4 Modena Local Health Authority, AUSL Modena, Modena, Italy; 5 Medicine and Surgery, University of Parma, Parma, Italy; 6 Department of Life Health Sciences and Health Professions, Link Campus University, Rome, Italy

**Keywords:** Adult palliative care, Hospice and Palliative Care Nursing, Caregivers, Stress, Psychological, Caregiver Burden, Dementia

## Abstract

**Abstract:**

**Introduction:**

Spirituality has gained increasing attention in healthcare, particularly in palliative care, as it supports meaning, purpose and connection during illness. While literature extensively explores patients’ spiritual needs, growing evidence highlights the importance of addressing the spiritual needs of caregivers and healthcare professionals. Caregivers and relatives often face emotional, ethical and practical challenges during prolonged care pathways, impacting their well-being. Limited training and tools can hinder spiritual care, contributing to distress and burnout among professionals and unmet needs for families. Addressing spirituality in neurodegenerative disease palliative care is essential for holistic, person-centred approaches that foster coping, hope and ethical decision-making. This scoping review aims to map evidence on spiritual needs and challenges of caregivers, relatives and healthcare staff in end-stage neurodegenerative disease care.

**Methods and analysis:**

This review will follow the Joanna Briggs Institute framework (JBI) for scoping reviews. The search and reporting process will be guided by the Preferred Reporting Items for Systematic Reviews and Meta-Analyses Extension for Scoping Reviews checklist. Inclusion criteria followed JBI’s population, concept and context framework with no date or geographical limits; only English and Italian sources ensured accurate interpretation. Searches will use university-access databases and grey literature to capture policy and non-peer-reviewed sources. Databases selected: PubMed, CINAHL, APA PsycINFO and Scopus for comprehensive, multidisciplinary coverage. This inclusive approach is aligned with the purpose of scoping reviews, which aim to map the breadth and depth of available literature. Researchers independently screened titles/abstracts in Rayyan software with blinding, resolved discrepancies collaboratively, piloted and refined extraction tables, and jointly synthesised themes through iterative meetings to ensure rigour and consensus. Findings will highlight existing knowledge, identify gaps and inform future research and practice.

**Ethics and dissemination:**

Since secondary data will be analysed, no ethical approval is required. The results will be disseminated through publications subject to peer review. The protocol has been registered with the Open Science Framework https://doi.org/10.17605/OSF.IO/X9275

**STRENGTHS AND LIMITATIONS OF THIS STUDY**

- Rigorous methodological framework: The review will follow a well-established scoping review methodology to ensure a high-quality and transparent process.
- Comprehensive and inclusive search strategy: Searches will be conducted across four disciplinary and cross-disciplinary databases, supplemented by grey literature to minimise publication bias and enhance comprehensiveness.
- Collaborative and internationally informed approach: The review team includes researchers and specialists engaged with an international network specialising in palliative and spiritual care research and clinical practice, ensuring relevance and global perspective.
- Shared software: The use of Zotero and Rayyan allows for efficient working across regions and countries.

## Introduction

 Spirituality is a multifaceted concept that has undergone numerous definitions and transformations, often in secular terms. The WHO^1^ recognises spirituality as one of the fundamental dimensions of health. Research indicates that spirituality—with or without religious affiliation—can positively influence well-being by enhancing self-esteem, compassion, emotional resilience and the capacity to address existential questions.^2^ It is widely acknowledged as a core aspect of human experience, with the potential to profoundly affect overall well-being, particularly during advanced illness.^3^ International studies further demonstrate that spirituality can shape health outcomes and provide comfort, meaning and connection during life’s most vulnerable stages.^4^ Conversely, one major barrier to the implementation of spiritual care is the lack of a shared understanding and clear definition of spirituality among healthcare professionals, compounded by difficulties in distinguishing it from religion or psychological support.^5^ In traditional Western hospital settings, spiritual care is often confined to religious representatives or chaplaincies.^6^ The complexity and overlapping nature of spiritual concepts have hindered the development of standardised frameworks, resulting in the marginalisation of spiritual and religious dimensions in everyday healthcare practice.^7 8^ Consequently, staff may remain unaware of the potential benefits of spiritual care for both patients and themselves. While spiritual care is essential for individuals facing existential struggles and seeking meaning in the face of illness, it also holds value for healthcare professionals, family members and caregivers. However, despite such ongoing debate and interest, the incorporation of spiritual care into clinical practice—particularly for staff and carers working or supporting individuals in palliative care for neurodegenerative conditions—remains uneven and underdeveloped.

### Background

In recent years, interest in spirituality has grown across diverse fields, reflecting cultural, religious and societal changes. This trend, particularly evident in Western societies, marks a shift from institutionalised religion towards a more individualised search for meaning, self-understanding and connection with others.^9^ Spirituality—whether linked to personal beliefs or broader existential perspectives—has become increasingly relevant in healthcare, especially in palliative care, by improving the overall quality of care of the person. The European Association for Palliative Care (EAPC) highlighted the importance of spirituality by giving a definition as ‘the dynamic dimension of human life that relates to the way people/community experience, express and/or seek meaning, purpose and transcendence, and the way they connect to the moment, to self, to others, to nature, to the significant and/or the sacred’.^4^ Kukulka *et al*^10^ and Robinson and Holloway^11 12^ highlight how the early integration of palliative care can improve the management of physical symptoms and address patients’ spiritual and psychological needs. In line with this perspective, Kluger *et al*^13^ emphasise the importance of a personalised palliative approach that can address the clinical and ethical challenges arising in the care of progressive neurological disorders. Evidence suggests that spirituality helps individuals cope with the turmoil associated with end-of-life by fostering a sense of meaning, purpose, hope and guidance.^10 14^ Patients with end-stage dementia who were referred for palliative care consultation during hospitalisation were more likely to receive treatment for physical symptoms, have their spiritual needs met (47%) and receive palliative care (25%).^15^

Spirituality manifests as a need for connection—with the patient, their family and the transcendent. In palliative care and neurodegenerative diseases, this need is expressed in a profound and complex way. Although the literature has extensively explored patients’ spiritual needs, a growing body of research is highlighting the importance of also considering the spiritual needs of those who provide care—healthcare professionals and caregivers.^314 16^,^18^ Relatives and caregivers are often involved in intensive, prolonged care pathways and face emotional, ethical and caregiving challenges that can have a profound impact on their well-being.

Healthcare professionals frequently encounter suffering, death and vulnerability, prompting them to reflect on the meaning of their work and the significance of care. They report a need for time and space to listen, as well as for empathetic relationships that extend beyond mere technical gestures. The lack of specific training on what spirituality and spiritual care mean, along with the absence of shared tools, hinders the integration of this dimension into clinical practice. This gap can lead to the emergence of spiritual distress, particularly among care professionals who often confront the sense of helplessness in the face of patient suffering. Such experiences may be accompanied by feelings of frustration, powerlessness and burnout, which can compromise the quality of relationships and care.^3 10 11 19 20^ Similarly, caregivers experience an often-invisible care burden that includes not only the practical management of illness but also the emotional and spiritual support of their loved one. Family members express significant spiritual needs such as continuous presence, spiritual accompaniment even after death, during bereavement and remembrance; they feel the need to share their pain, receive comfort and spiritual support, and seek reconciliation with themselves and others, as well as inner peace and acceptance of the end.^17 19 21^ Baumgardner and Mayo^16^ explored the lived experience of spiritual well-being among family caregivers of people with dementia in palliative care, highlighting how spirituality can serve as a crucial resource for coping with pain, loneliness and the sense of loss, where spiritual well-being can help alleviate anxiety, social isolation and the high caregiving burden experienced. Recent studies have further examined these aspects; Brandstötter *et al*^17^ identified the spiritual needs of people with amyotrophic lateral sclerosis (ALS) and their caregivers, emphasising the importance of personalised spiritual support. As noted by Paal and Lorenzl,^3^ spirituality is rarely promoted as an integral component of health and well-being in healthcare settings. This can lead to confusion, as patients and caregivers often do not understand or express their spiritual needs effectively. Integrating the spiritual dimension into healthcare—especially in the context of neurodegenerative diseases—represents a key element in ensuring a person-centred approach that addresses existential experiences.

In many cases, caregivers face questions about the meaning of illness, the progressive loss of patient autonomy and their own role; similar to their loved ones, caregivers experience increasing levels of dependency, physical care demands and the need for long-term life adjustments.^10 22^

The literature suggests several interventions to support caregivers in addressing the spiritual needs and challenges of people with neurodegenerative diseases. Among these, the importance of ensuring access to experienced spiritual counsellors,^18^ the availability of specialised palliative care teams dedicated to the complex needs of patients and families, and spaces where issues such as religion and spirituality can be addressed—alongside physical symptoms, social support and caregiver burden assessment—are emphasised.^310^,^12^

Yet, despite this recognition, spiritual care is often under-addressed in clinical settings, where time constraints, lack of training and the absence of shared tools pose significant barriers.^19^ Recent studies have begun to explore the role of spirituality in neurodegenerative conditions, showing that early integration of palliative care can support not only physical symptom control but also patients’ spiritual and psychological needs.^10^,^12^ This is particularly relevant in progressive neurological diseases, where ethical and existential challenges are common, and a personalised approach is essential.^13^ Moreover, spirituality has been shown to help individuals with advanced dementia cope with emotional distress, fostering hope and meaning,^14^ while those receiving palliative counselling are more likely to have their spiritual needs addressed.^15^ However, the spiritual dimension of care concerns not only patients.

This scoping review is therefore necessary to map the existing evidence on spirituality in palliative care for neurodegenerative diseases, with particular attention to the experiences and needs of caregivers and relatives. It aims to clarify how spirituality is understood and addressed in these contexts, identify gaps in practice and research, and support the development of more holistic, inclusive models of care.

## Methods and analysis

A scoping review will be conducted to identify knowledge gaps and explore potential opportunities for further research regarding the spiritual needs and challenges experienced by healthcare staff and relatives caring for adults with end-stage neurodegenerative diseases. This review is guided by the Joanna Briggs Institute (JBI) methodology and recommendations.^23 24^ According to Munn and Peters, scoping reviews are particularly useful for clarifying key concepts and definitions in the literature, identifying gaps in the evidence and providing an updated overview for healthcare professionals, researchers and educators to inform future research and practice development.^23 25^ The search and reporting process will be guided by the Preferred Reporting Items for Systematic Reviews and Meta-Analyses Extension for Scoping Reviews (PRISMA-ScR) checklist.^26 27^ The scoping review will commence in winter 2025, with completion in summer 2026.

### Review question

This review is based on the work of an international, diverse group of professionals, including experienced researchers, clinical nurses and academics specialising in palliative and spiritual care. Some members have an extensive publication record in this field, contributing to the evidence base on spirituality and end-of-life care for many years. Others bring valuable hands-on expertise from clinical practice in palliative care units and neurodegenerative disease centres, ensuring that the research questions are inspired and grounded in real-world challenges and patient needs. The review question is framed using the Participants, Concept and Context framework (table 1), as recommended by the JBI^3 24^:

**Table 1:** PCC framework

	Research question	Keywords
Participants	Healthcare staff and relatives caring	“Health Personnel”[MeSH] “Family”[MeSH] “Caregivers”[MeSH]
Concept	Spiritual needs	Spirituality [MeSH]
Context	End-stage neurodegenerative diseases	“Neurodegenerative Diseases”[MeSH]

MeSH, Medical Subject Headings.

‘What is known from the existing literature about the spiritual needs and challenges (Concept) experienced by healthcare staff and relatives (Participants) caring for adults with end-stage neurodegenerative diseases (Context)’?

The following subquestions will be developed to guide the study:

1. What types of spiritual needs are reported by healthcare staff and relatives in the context of end-stage neurodegenerative diseases?
2. What spiritual challenges do healthcare staff and relatives encounter while providing care to individuals with end-stage neurodegenerative diseases?
3. What overarching strategies are described in the literature to support healthcare staff and relatives in addressing spiritual needs and challenges?
4. What specific practices and interventions are reported in the literature to assist healthcare staff and relatives in responding to spiritual needs and challenges?
5. What contextual factors (eg, cultural, ethical, organisational) influence the recognition and management of spiritual needs in this care setting?

### Search strategy

This scoping review addresses a relatively new and evolving topic. Following an initial exploratory review, the research team agreed not to restrict the search by publication date in order to capture the widest possible range of evidence and perspectives. This inclusive approach is aligned with the purpose of scoping reviews, which aim to map the breadth and depth of available literature. The chosen search interface was selected based on its reliability and availability through the university’s institutional access, ensuring consistency and accessibility for the research team. Given that the topic of spirituality in palliative and end-of-life care continues to be debated and shaped by both national and international organisations, the inclusion of grey literature was deemed essential. This allows the review to incorporate policy documents, reports and other non-peer-reviewed sources that may offer valuable insights not captured in academic databases. Database selection was guided by relevance and scope in relation to the research topic: PubMed was included for its comprehensive coverage of biomedical and health sciences literature. CINAHL Plus with Full Text was selected to access nursing and allied health research. APA PsycINFO was chosen for its focus on psychological and behavioural sciences. Scopus was added to ensure multidisciplinary coverage and access to international literature, including conference proceedings, and to support citation tracking.

### Inclusion criteria

The inclusion criteria for this scoping review support the collection and extraction of data relevant to the research question and objectives. Following the JBI construct of population, concept and context, the inclusion criteria are in table 2.

**Table 2:** Inclusion/exclusion criteria

	Inclusion criteria	Exclusion criteria
Participants	Studies focusing on healthcare staff (eg, nurses, physicians, allied health professionals), caregivers and relatives (eg, family members, close friends) providing care or support to adults with end-stage neurodegenerative diseases.	Studies focusing exclusively on patients with neurodegenerative diseases, without reference to caregivers or healthcare staff. Studies involving paediatric populations or caregivers of children/adolescents.
Concept	Studies exploring spiritual needs, spiritual challenges, spiritual distress or spiritual support experienced by healthcare staff and/or relatives. Studies discussing the integration of spiritual care into caregiving practices.	Studies focusing solely on religious practices without reference to broader spiritual dimensions. Research addressing general psychological or emotional needs without specific reference to spirituality.
Context	All care settings where adults with end-stage neurodegenerative diseases are treated (eg, hospitals, hospices, long-term care facilities, home care). Studies conducted in any geographical location or cultural context.	Studies conducted in experimental or non-clinical settings (eg, simulation labs, educational settings without direct caregiving). Research not involving end-stage disease phases (eg, early or midstage neurodegenerative conditions). Studies focusing on general chronic illness without specific reference to neurodegenerative diseases.

This scoping review will include a broad range of evidence sources to ensure comprehensive coverage of the topic. These sources will include quantitative, qualitative and mixed-methods studies, regardless of methodological approach. This scoping review will include a wide range of primary evidence sources, but systematic reviews and other review types will not be included as main sources of evidence. However, relevant reviews identified during screening may be used for contextual background or discussion, and any systematic reviews deemed useful will be used for reference checking to ensure important primary studies are not missed. Therefore, no limits will be placed on publication date or geographical location. The search will be limited to articles published in English and Italian, with no date restrictions. The language restriction to English and Italian was applied to ensure accurate interpretation by the research team.

In accordance with the Medical Subject Headings (MeSH) terms and keywords used in the search strategy, the review will include the following categories of neurodegenerative diseases: motor neuron diseases (including ALS), Parkinson’s disease and related parkinsonian disorders, neurodegenerative dementias (such as Alzheimer’s disease, frontotemporal dementia and dementia with Lewy bodies), Huntington’s disease, hereditary ataxias and other chronic progressive neurodegenerative or neuromuscular conditions captured by our search terms.

### Query strings

The search strategy was collaboratively developed by the research team in consultation with the library service of the University of Birmingham (UK). The final search strategy was initially constructed for PubMed (table 3) and subsequently adapted for use across the other databases. MeSH terms were translated into CINAHL Headings for the search in CINAHL Plus with Full Text, and into the APA Thesaurus of Psychological Index Terms for the search in APA PsycINFO via EBSCOhost. For Scopus, only free-text terms were used. A language limit will be applied to include only studies published in English and Italian. No other search limits will be imposed, to minimise the risk of inadvertently excluding relevant literature. All potential papers will be exported to the reference manager Zotero, where full-text copies can be imported by any team member and from which the reference list will be generated (see https://www.zotero.org/).

**Table 3:** Search strategy and query string for one of the databases

Database	Search query	Result
PubMed	Population	
#1	(“Health Personnel”[MeSH]) OR (“Healthcare workers”[Title/Abstract] OR “Healthcare staff”[Title/Abstract] OR Nurse*[Title/Abstract] OR Physician*[Title/Abstract]) OR (“Family”[MeSH]) OR (“Caregivers”[MeSH]) OR (Relative*OR Caregiver*OR “family caregivers”[Title/Abstract])	
	Concept	
#2	(“Spirituality”[MeSH]) OR (spirituality [Title/Abstract] OR “spiritual sensitivity”[Title/Abstract] OR spiritual [Title/Abstract] OR “Spiritual Care” [Title/Abstract] OR “Spiritual Distress” [Title/Abstract] OR “Spiritual Need” [Title/Abstract])	
	Context	
#3	(“Neurodegenerative Diseases”[MeSH]) OR (“Motor Neuron Disease”[MeSH]) OR (“Neuromuscular Diseases”[MeSH]) OR (“Parkinson Disease”[MeSH]) OR (“Dementia”[MeSH]) OR (neurodegenerative[Title/Abstract] OR amyotrophic[Title/Abstract] OR Parkinson[Title/Abstract] OR dementia[Title/Abstract] OR “neurologic disorder”[Title/Abstract])	
	#1 AND #2 AND #3	277
LIMITS APPLIED	Language: English, Italian Other limits: None (no date or study-type filters applied)	

### Grey literature

This scoping review will adopt an inclusive approach to capture the full range of existing knowledge and perspectives on the spiritual needs and challenges experienced by healthcare staff and relatives caring for individuals with end-stage neurodegenerative diseases. Grey literature will be accessed through platforms such as: Google Scholar and OPEN Grey (DANS Data Stations); ProQuest Dissertations & Theses Global; Professional Networks and social media (Research Gate, International Palliative Care Organisation documents and LinkedIn).

### Screening and data extraction

The screening procedures for this scoping review are designed to uphold scientific rigour while remaining pragmatic and feasible for the research team. The process is structured in multiple rounds, beginning with independent title and abstract screening conducted by each researcher using the Rayyan platform.^28^ Blinding is enabled during this phase to reduce bias and ensure that inclusion decisions are made without influence from other reviewers. All identified records will be imported into the web-based bibliographic management tool Rayyan, which will facilitate the removal of duplicate entries. Following deduplication, the title and abstract screening phase will be conducted within Rayyan. This initial screening will be conducted by two pairs of researchers, with each pair reviewing the list of articles from opposite ends (ie, one pair from the top down and the other from the bottom up) to ensure thoroughness and minimise bias. All screening decisions will be made collaboratively by the researchers. Decisions to include, exclude or flag studies for discussion will be based strictly on the predefined inclusion and exclusion criteria for the scoping review. Studies that do not meet these core criteria—such as lacking relevance to the population or conceptual focus—are excluded. Any discrepancies between the two researchers during the full-text screening will be resolved through discussion, with the involvement of a third reviewer if necessary. The outcomes of the search and the study selection process will be thoroughly documented in the final scoping review and illustrated using a PRISMA-ScR flow diagram. All screeners were introduced to the screening instructions through a combination of research team meetings, email communications and access to a recorded briefing session. One researcher with prior experience using the Rayyan platform provided guidance to the team, outlining the screening process and establishing clear procedural steps. This preparatory phase ensured a consistent, thorough approach to title and abstract screening, supporting accuracy and efficiency throughout the review.

Scoping reviews seek to comprehensively map out a body of literature; therefore, they do not include a step for the assessment of the methodological quality of the studies or the risk of biases of sources of evidence. For the data extraction, a table developed ad hoc by the team will be used, consistent with the research questions and inclusion criteria. This table will have a general part which includes information such as year, country, aim, study design, study participants, context, key findings, median age and characteristics. A more specific table will be used, with further information such as types of spiritual needs, strategies or interventions described, spiritual challenges of healthcare staff and relatives, contextual factors and outcome. The extraction phase will begin with a pilot test of the data extraction tool to ensure clarity, consistency and alignment with the review’s aims. This initial calibration exercise will involve two reviewers independently extracting data from a small sample of studies (approximately 5–10). Following this, the reviewers will meet to compare results, discuss discrepancies and refine the extraction form as needed. If an important level of agreement is not achieved, further adjustments will be made to improve reliability. All modifications to the tool will be documented and reported in the final review. Once the tool has been validated, the full extraction process will proceed with researchers independently reviewing all included articles. To support consistency and interpretive integrity, reconciliation sessions will be scheduled at key points—midway and at three-quarters of the extraction phase—allowing the team to address any emerging issues, harmonise interpretations and refine category development. On completion of the extraction, a comprehensive data table will be compiled. This table will serve as the foundation for the synthesis phase, supporting the mapping of conceptual categories and the identification of patterns, gaps and thematic concentrations across the literature. All researchers were introduced to the extractor instructions through a combination of research team meetings, email communications and access to a recorded briefing session. One researcher with prior experience using the Rayyan platform provided guidance to the team, outlining the extractor process and establishing clear procedural steps. This preparatory phase ensured a consistent and thorough approach to data extraction, supporting both accuracy and efficiency throughout the review.

### Analysis of evidence

The synthesis process in a scoping review shifts toward organising and interpreting the collected information in a way that meaningfully addresses the research question. Rather than aggregating data statistically, the aim is to construct a descriptive and conceptual overview of the existing literature. This involves identifying patterns, recurring themes and conceptual groupings that reflect the scope and diversity of the studies included.

1. The extracted data are reviewed and interpreted collaboratively by the research team, allowing for multiple perspectives to inform the development of thematic categories. These categories help illuminate how the literature frames the topic—in this case, the spiritual needs and challenges of caregivers and relatives supporting individuals with neurodegenerative end-stage conditions. Through this process, the team examines how numerous studies conceptualise care, spirituality and relational dynamics, while also identifying gaps, inconsistencies or under-represented areas.
2. Although formal bias assessment tools are not typically applied in scoping reviews, the team remains attentive to the limitations of individual studies and the synthesis itself. This includes reflecting on methodological diversity, clarity of reporting and relevance to the review’s aims. Where ambiguity arises, particularly in terminology or scope, the team engages in dialogue to ensure interpretive integrity and transparency. Rather than applying rigid evaluative frameworks, the synthesis is guided by transparency, relevance and the integrity of interpretation.
3. Ultimately, the synthesis provides a structured narrative that maps the landscape of existing knowledge, offering insight into how the topic has been studied, where further inquiry is needed and how future research might build on these foundations.

To ensure reliability in the synthesis phase, the process will be conducted collaboratively by a team of five researchers. Each team member will begin by independently reviewing the extraction tables and identifying preliminary conceptual categories based on the data. This initial phase enables diverse interpretations and mitigates individual bias. The team will then convene for an inter-synthesis meeting, where initial findings, emerging hypotheses and thematic groupings will be shared and discussed. This dialogue serves to refine understanding, challenge assumptions and align perspectives. Following this, researchers will continue working independently to deepen and consolidate the categories before reconvening to finalise a coherent and collectively agreed framework. This iterative and dialogic approach ensures both methodological rigour and interpretive integrity throughout the synthesis process.

Regular inter-synthesis meetings will be planned to share, compare and refine the emerging categories. These sessions serve as a space for critical dialogue, where each category or interpretive issue that does not align across researchers is openly discussed. In cases where consensus cannot be reached internally, an external researcher will be invited to provide a third opinion. This reconciliation procedure ensures that interpretive decisions are made with integrity, striking a balance between individual insight and collective agreement. Through this iterative process—moving between independent analysis and team-based synthesis—the research team will arrive at a coherent and well-founded set of conceptual categories. This process reinforces transparency and supports the development of a coherent and trustworthy synthesis framework.

This synthesis approach is aligned with the JBI methodology for scoping reviews,^29^ employing iterative data charting, collaborative interpretation and thematic/narrative synthesis to ensure transparent and rigorous evidence mapping.

### Patient and public involvement

This review will not involve patient and public involvement at any stage. The completed review will be disseminated through publication, and the findings may be of value to health and social care researchers.

## Ethics and dissemination

This review will involve secondary analysis of published literature; therefore, ethics approval is not required. However, the completed review will be submitted for publication in a peer-reviewed journal and presented at relevant international conferences. It is anticipated that the published findings will also be used for educational purposes within nursing and midwifery curricula, particularly in courses addressing palliative, spiritual care and neurodegenerative diseases. Throughout the conduct of this scoping review, we will actively engage with members of international groups focused on informing evidence-based strategies for integrating spiritual care within palliative care frameworks (eg, EAPC, Italian Society of Palliative Care -SICP). This will ensure alignment with current global priorities and foster dialogue across disciplines. The scoping review will also support the creation of a research, policy and practice-oriented group that will contribute to the development of future research proposals.
