# Supplementary material for: Spiritual care challenges and needs of carers, health professionals and relatives of individuals living with neurodegenerative diseases in palliative and end-of-life care: a scoping review protocol
Source: BMJ Open. 2026 Apr 2;16(4):e114259. doi: 10.1136/bmjopen-2025-114259 (PMC13052809; doi:10.1136/bmjopen-2025-114259)
Supplement: Supplementary file 1 [file bmjopen-2025-114259.reviewer_comments.pdf]

PEER REVIEW HISTORY

BMJ Open publishes all reviews undertaken for accepted manuscripts. Reviewers are asked to complete a checklist review form and are provided with free text boxes to elaborate on their assessment. These free text comments are reproduced below.

ARTICLE DETAILS

Title (Provisional)

Spiritual care challenges and needs of carers, health professionals, and relatives of individuals living with neurodegenerative diseases in palliative and end-of-life care: a scoping review protocol.

Authors

De Luca, Enrico; Saba, Andreina; Bertarini, Laura; Brusini, Antonio; Artioli, Giovanna; Dellafiore, Federica

---

VERSION 1 - REVIEW

**Reviewer**	**1**
**Name**	**Giordano, Andrea**
**Affiliation**	**Fondazione IRCCS Istituto Neurologico Carlo Besta, Neurology, Public Health and Disability Unit**
**Date**	**10-Jan-2026**
**COI**	**None**

The present manuscript is a scoping review protocol which aims to map the evidence on spiritual care challenges and needs of carers, health professionals, and relatives of individuals living with neurodegenerative diseases in palliative and end-of-life care.

I found the protocol very interesting, well-written, clear in the objectives and methods. Also, I believe that the results of the review will provide interesting data to the international palliative care community.

I have a few points which should be better clarified:

- I have found a bit confusing the use of terms ‘relatives’ in the title, ‘caregiver’, and other similar terms throughout the manuscript. I would suggest authors choose and clarify why they chose a particular term, e.g. informal caregiver, and be consistent with it throughout the document. Notably, in Table 1, they have reported ‘caregivers’ as Mesh to be searching for in the databases.

- I would suggest authors to clarify which neurodegenerative diseases would include in the scoping review.

- Objective 3: I would suggest authors split 'strategies' from 'interventions'. In my view, they are not the same concept, and therefore should be two reported as two different objectives. Do you expect to find relevant interventions for healthcare professionals and /or caregivers?

- I would also suggest clarifying which study design(s) would consider in the review and whether they include (systematic) reviews in the scoping review.

- Analysis of evidence: Even if it is clear to me how they will perform the data synthesis, I would suggest authors add a reference to a theoretical framework. As such, it seems that it is a spontaneous ad hoc effort by the authors themselves.

VERSION 1 - AUTHOR RESPONSE

Reviewer 1 comments	Authors response
I have found a bit confusing the use of terms 'relatives' in the title, 'caregiver', and other similar terms throughout the manuscript. I would suggest authors choose and clarify why they chose a particular term, e.g. informal caregiver, and be consistent with it throughout the document. Notably, in Table 1, they have reported 'caregivers' as Mesh to be searching for in the databases.	Thank you for this valuable comment regarding term consistency.<br><br>We reviewed the use of the terms caregivers and family throughout the manuscript and made amendments where necessary.<br>In line with Table 1, we decided to remove the term informal caregivers from the text and replace it with relatives.
- I would suggest authors to clarify which neurodegenerative diseases would include in the scoping review.	In accordance with the MeSH terms and keywords used in the search strategy, the review includes the following categories of neurodegenerative diseases: motor neuron diseases (including amyotrophic lateral sclerosis), Parkinson's disease and related parkinsonian disorders, neurodegenerative dementias (such as Alzheimer's disease, frontotemporal dementia and dementia with Lewy bodies), Huntington's disease, hereditary ataxias, and other chronic progressive neurodegenerative or neuromuscular conditions captured by our search terms.
- Objective 3: I would suggest authors split 'strategies' from 'interventions'. In my view, they are not the same concept and therefore should be two reported as two different objectives. Do you expect to find relevant interventions for healthcare professionals and /or caregivers?	Thank you for this helpful comment.<br><br>We have now separated strategies and interventions to reflect their different meanings, and we have revised the manuscript accordingly.

	As a result, we have included two distinct review questions to address these concepts more clearly.
- I would also suggest clarifying which study design(s) would consider in the review and whether they include (systematic) reviews in the scoping review	We have clarified in the manuscript that this scoping review will include "a broad range of evidence sources to ensure comprehensive coverage of the topic" and added "These sources will include quantitative, qualitative, and mixed-methods studies, regardless of methodological approach".<br><br>We also have added, "other reviews—systematic or otherwise—will not be included as primary sources of evidence. During the screening process, such reviews may still be examined by the team to determine whether they offer additional value for contextual background or for informing the discussion.<br>In addition, any systematic reviews identified during screening and considered relevant will be used for reference checking to ensure that key primary studies are not overlooked".
- Analysis of evidence: Even if it is clear to me how they will perform the data synthesis, I would suggest authors add a reference to a theoretical framework. As such, it seems that it is a spontaneous ad hoc effort by the authors themselves.	Thank you,<br>we added a reference of the framework we used for the synthesis of evidence (JBI).
If you have selected 'Yes' above, please provide details of any competing interests.: not applicable	Not clear this comment to whom belong to. However, we have checked the competing interest section and it seems filled in correctly.
